# A group study on the effects of a short multi-domain cognitive training in healthy elderly Italian people

**DOI:** 10.1186/s12877-018-1014-x

**Published:** 2018-12-27

**Authors:** Chiara F. Tagliabue, Sabrina Guzzetti, Giulia Gualco, Giovanna Boccolieri, Alfonsa Boccolieri, Stuart Smith, Roberta Daini

**Affiliations:** 1 Center for Mind/Brain Sciences (CIMeC), University of Trento, Rovereto, Italy; 2 Department of Psychology, University of Milano-Bicocca, Milan, Italy; 3 Memory clinic, Neurology Service, Humanitas San Pio X, Milan, Italy; 4 RSA Sant’Andrea, Monza, Italy; 5 Associazione Alzheimer Monza e Brianza, Monza, Italy; 6 Southern Cross University, Coffs Harbour, NSW Australia; 7 NeuroMI - Milan Center for Neuroscience, Milan, Italy; 8 Psychology Department, University of Milano - Bicocca, Milan, Italy

**Keywords:** Active ageing, Cognitive stimulation, Training intensity

## Abstract

**Background:**

Alongside physiological cognitive ageing, nowadays there is an alarming increase in the incidence of dementia that requires communities to invest in its prevention. The engagement in cognitively stimulating activities and strong social networks has been identified among those protective factors promoting successful cognitive ageing. One aspect regarding cognitive stimulation concerns the relevance of the frequency of an external intervention. For this reason, the aim of this study was to evaluate the efficacy of a 3-month cognitive training program, once per week, in a group of healthy elderly aged over 60 years old. Their results were compared with those of a passive control group.

**Methods:**

The training consisted of a weekly session of multi-domain and ecological cognitive exercises performed in small homogenous (i.e. same cognitive level) groups. The scores obtained in a neuropsychological assessment by the experimental and control groups were compared at pre- and post-training. In addition, by means of a questionnaire, we also evaluated the indirect effect of the program on participants’ mood, socialization and perceived impact on everyday activities.

**Results:**

Overall, the experimental group showed a general improvement in cognitive functioning following the training program, even with the frequency of once per week. Greater improvements were observed mainly on executive functions and short-term memory, but general cognitive functioning and non-verbal reasoning also showed a tendency to an improvement. It is noteworthy that a majority of the participants reported to have subjectively experienced an improvement in their everyday life and a positive influence on both mood and socialization.

**Conclusions:**

These results show that even a low-intensity training program is able to promote some of the protective factors that support successful cognitive ageing. Moreover, this multi-domain approach proved to be an excellent training method to transfer gains not only to other cognitive domains, but also to everyday living.

**Trial registration:**

NCT03771131; the study was retrospectively registered on December 7th 2018.

**Electronic supplementary material:**

The online version of this article (10.1186/s12877-018-1014-x) contains supplementary material, which is available to authorized users.

## Introduction

Recent advances in our understanding of the ageing process, as well as improvements in health and wellness monitoring technologies, nurture the hope that older adults will be increasingly empowered in order to remain independent and benefit from a high quality of life. For these reasons, the challenge of our time is to provide older adults with the opportunity to avoid social isolation, loss of interest/contact in the world and decline in social, emotional, physical and cognitive stimulation.

Although cognitive decline affects the whole older population, it can develop and evolve in very quantitatively and qualitatively different ways. Physiologically, ageing is associated with a reduction in information processing speed, learning abilities and memory, and with a decline in the executive function (including reasoning, problem solving, planning and mental flexibility; for a review, see [1]. Despite a deterioration of these cognitive abilities, commonly labelled as “fluid intelligence”, knowledge or the so called “crystallized intelligence” [2] is maintained or even increases over time [3].

However, beyond physiological ageing, the alarming increase in the incidence of dementia is considered by some as ‘the global challenge of 21st century’ [4]. Indeed, in 2005 a worldwide epidemiological study [5] estimated that around 24.3 million people were suffering from dementia and that 4.6 million new cases are diagnosed every year, with the prediction of up to 81.1 million cases by 2040. In Italy specifically, the incidence of dementia is around 1% among people over 60 years, a percentage that is bound to increase with increasingly older societies [6]. It is therefore unavoidable that the ageing of the population, together with the consequential increase in the incidence of dementia, will have an impact on the costs that national health services must respond to [7]. For this reason it is necessary that societies invest in evidence-based programs of dementia prevention.

Primary prevention, by enhancing protective factors and decreasing risk factors, has also started to gather growing interest in the field of cognitive functioning. In particular, exploration of both lifestyle and environmental factors that affect the health of our brain are of interest [8]. Indeed, controlling for vascular risk factors, engagement in intellectually stimulating activities, increased engagement in physical exercise and social involvement, a balanced diet and early detection of depression symptoms are some of the main factors identified as promoters of successful cognitive ageing (for a review, see [9, 10]). Such protective behaviours not only improve our cognitive health by inducing brain plasticity [11], but, more importantly, they can even prevent or delay the expression of clinical dementia by contributing to the formation of a larger so-called cognitive reserve [12]. Cognitive reserve is a concept that accounts for the individual differences in cognitive functioning, acting as a mediator between degree of pathology and its manifestation.

Taken together these findings highlight the need for effective techniques that can actually contrast the cognitive decline due to ageing. In this respect, there is now a growing body of evidence showing how cognitive training interventions in healthy older adults have beneficial effects in maintaining or increasing cognitive functioning (e.g. [13, 14]). However, to be considered truly effective, cognitive training programs have to meet a series of criteria. Not only should these interventions enhance the subject’s performance in the trained cognitive task, but any observed improvement should also be maintained over time, transferred to other tasks engaging the same or other cognitive functions and, hopefully, improve everyday living. This last goal has proven to be difficult to achieve due to the lack of appropriate measures of everyday cognitive functioning (e.g. [15]) in healthy older adults, although some new protocols have been developed [16].

In order to fulfil the above-mentioned criteria, different behavioural-experimental approaches have been proposed over the last few years [17], including strategy, multimodal and process training protocols, each one of them having their own advantages and disadvantages. In strategy-based training, subjects are trained on specific strategies that may help increase their performance in a given task, for example the method of loci as in memory trainings [18]. They often show large and long-lasting benefits on the trained task, but limited transfer. On the other hand, multimodal interventions (e.g. [19, 20]) are usually more complex and socially engaging approaches. They can vary from learning new activities, such as gardening or photography, to physical exercises or voluntary work. Their positive effects can be widespread and due to their social nature they may encourage participants to keep practising even after the training finishes. Unfortunately, such approaches to training show relatively small transfer to other domains and it is sometimes difficult to pinpoint which aspect of the training actually brings benefit. Finally, process-based methods consist of exercises designed to train a highly specific cognitive function without necessarily relying on explicit strategies (e.g. [21] for visual search). These interventions aim to transfer potential positive effects to untrained tasks that engage the same cognitive process. This approach requires a careful analysis of the proposed exercises to ensure maximum transfer effects on untrained task conditions. To date, the latter method appears to be the most promising intervention, as several studies [22–24] have reported long-lasting performance enhancement across different tasks. However, the major limitation of a single-domain approach is that it underestimates the importance of interactions between multiple mental functions, which is fundamental in everyday activities [25].

To overcome these limitations, multi-domain approaches to training aim at emphasizing complex cognitive interactions by simultaneously engaging either multiple lower-level mental processes (such as attention, perception, memory, etc.) or higher-level executive functions (such as inhibition, flexibility of thinking, problem solving, planning, etc.). As a result, experimental studies using multi-domain programs reported stronger effects on benefit maintenance at follow-up [26] and more advantages in the far transfer of the benefits [27, 28]. In addition to the constraint that mental functioning enhancement might occur only if the proposed tasks are cognitively challenging [29], the optimal amount of cognitive engagement required will likely differ on an individual basis and be intrinsically related to an individual’s previous experiences, fields of expertise and current cognitive status. It is therefore clear that a personalized approach to cognitive training might lead to more sustained and significant outcomes (e.g. [30]).

Based on these experimental findings, the main goal of the present study was to develop and validate a multi-domain cognitive training program for active ageing. We wondered whether one-day-a-week sessions, administered in small groups, would be stimulating enough to improve the cognitive performance of healthy elderly. Previous studies employing a multi-domain cognitive training performed more frequently either in small groups [26, 31] or individually at home [27] found that such approach was more effective in improving reasoning abilities [26], executive functions [31] and attention [27]. However, we were interested in a non-intense program for multiple reasons. Firstly, it has been shown that intensive training programs do not always yield better results (e.g. [32]), and starting with such a low training intensity would let us compare, in future studies, the effect of increasing the training dosage. Secondly, healthy individuals often have a busy life and this results in a lack of time or motivation to follow a more intensive program. Finally, if such a low intensity program is indeed effective, it could be easier to implement in reality despite insufficient resources in terms of both 1) spaces for delivering the program, and 2) costs for experienced neuropsychologists to manage groups and the training program. With this aim, we examined the effects of a 3-month training program consisting of 1-h weekly sessions of multi-domain and ecological (i.e. relating to everyday life demands) cognitive exercises, within a pre/post-test design and comparing the scores obtained by the participants to those of a passive control group. Brief psycho-educational interventions on general cognitive functioning were also provided throughout sessions, since some studies (e.g. [33]) showed that they aid in improving metacognition and coping strategies to face age-related changes in mental processes. Moreover, in order to tailor the training intervention to individuals, sessions were carried out in small homogeneous groups, sorted according to overall cognitive performance in the pre-training neuropsychological assessment. The choice of a group approach was also preferred to an individual one due to known positive relationships between cognition and social involvement [34], specifically higher mental functioning and slower rate of cognitive decline for older adults actively engaged in a high number of social networks. Moreover, group settings act as a boost for the efficacy of cognitive interventions (for a review, see [13]). In addition to the cognitive outcomes, we also evaluated the indirect effect of the program on participants’ mood, socialization, and perceived impact on everyday activities using a questionnaire in order to include a subjective functional outcome measure.

## Methods

### Participants

One hundred eight participants were recruited through advertisements in local newspapers and flyers, within a project led by the Associazione Alzheimer Monza e Brianza, in partnership with the Department of Psychology of the University of Milano-Bicocca. Participants were assigned to the experimental (EG) or control group (CG) based on their time of application to the program: the first subjects applying were assigned to the experimental program, while the others to the control group. Fifty-three participants were then assigned to the experimental training group, but only those who did not show a positive anamnesis for neurological and/or psychiatric diseases (*N* = 4) or with a suspected cognitive impairment, as assessed in the pre-training neuropsychological evaluation, or those who completed at least 11 h training (~ 85% attendance, in order to ensure continuity of the intervention; discarded participants = 19), were included in the analyses. Thirty individuals (21 women, 9 men; mean age ± standard deviation: 70.17 ± 6.46) formed the final experimental group. The other 55 participants were assigned to the control group, but only those who did not show a positive anamnesis for neurological and/or psychiatric diseases or with a suspected cognitive impairment (*N* = 1), as assessed in the first neuropsychological evaluation, or those who came back for the second assessment after a 3 months interval (54 out of 55), were included in the analyses. Fifty-three participants (34 women, 19 men; mean age ± standard deviation: 69.47 ± 6.39) formed the final control group.

All the participants gave their written informed consent to participate in the study. The project was approved by the Ethics Committee of the University of Milano-Bicocca and conducted in accordance with the 2013 Declaration of Helsinki.

### Neuropsychological assessment

Both experimental and control group underwent a neuropsychological assessment to investigate different cognitive domains, before and after (~ 3 months) the training program. Global cognitive functioning was evaluated through the Montreal Cognitive Assessment (MoCA; [35, 36]). For verbal and visuo-spatial memory, Forward and Backward Digit and Corsi Spans [37], the Short Story Test (two versions, [38, 39]; in the Short Story Test participants are required to remember immediately and after a 10-min delay a short story read by the experimenter. For each test we calculated the percentage of elements correctly recalled) and the Recall of Rey-Osterrieth Complex Figure (ROCF; [40]) were chosen. Visuo-constructional abilities were investigated by means of the ROCF Copy test [40]. The Semantic and Phonological Verbal Fluency Tests [38] and the Stroop Color and Word Test [41] were used to assess executive functions. Measures of attention were obtained through the Trail Making Test (TMT; [42]). Finally, non-verbal reasoning was assessed by means of Raven’s Coloured Progressive Matrices [43].

### Group sorting

Based on the scores obtained during the preliminary neuropsychological assessment, the participants of the experimental training group were divided into small subgroups (ranging from 8 to 12 subjects) to further address individualization of training.

For each individual of the experimental group, Z scores were calculated from the raw scores obtained on each neuropsychological test (based on the mean scores of the experimental group). Z scores were then averaged in order to obtain a composite global cognitive index for each subject. This global score was used as a reference for sorting participants into homogeneous groups, i.e. subjects with a similar global score were assumed to be at a similar cognitive level.

### Intervention

The experimental group attended weekly sessions of the multi-domain cognitive training. Each session lasted around 1 hour and the overall duration of the training was of 3 months, for a total of 13 sessions. Psychologists with a background in neuropsychology were responsible for designing the cognitive exercises and conducting the training sessions. All the exercises proposed were created with the program Microsoft Office PowerPoint (www.office.com), which proved to be a particularly effective format for presenting information to older adults [44], and displayed by means of a projector. Exercises were designed to stimulate different cognitive domains; some of them focused on one particular cognitive function (such as memory, attention, etc.), while others required the simultaneous engagement of different low-level cognitive functions or executive functioning. In order to provide an adequate level of difficulty and to avoid boredom, frustration and demotivation, all the exercises were adapted to the cognitive level of each group by changing some task parameters (e.g. speed, amount of stimulation) and trainers taught the groups strategies to perform the tasks more effectively. To promote improvement transfer to everyday life, some of the exercises proposed were ecological in their nature, in that, they asked participants to solve tasks that recalled everyday situations (such as remembering names or road maps). Trainers stimulated both individual work, by requiring each participant to give written answers, and teamwork, by encouraging the reciprocal exchange of different points of view among the group members.

Furthermore, throughout the training, approximately once every two sessions brief psycho-educational interventions focusing on how the brain works (including topics such as memory, attention or physiological and pathological ageing), and how healthy lifestyles (such as physical exercise and nutrition) may positively affect or preserve brain functioning were provided.

### Post-training questionnaire

At the end of the cognitive training, participants filled in a questionnaire (each question measured on a 5-point Likert Scale) assessing both the satisfaction level about the training and the possible impact of the program on participants’ everyday activities, mood and socialization (see Additional file 1 for a copy of the questionnaire).

### Statistical analyses

In order to verify the absence of differences at baseline between experimental and control groups, we performed t-test analyses for Age and Education Years, and also for the MoCA raw scores. This last measure, indeed, can be considered a measure of general cognitive functioning.

In order to verify whether there has been an overall impact of the training, we first ran a MANCOVA. In particular, it was computed to determine whether the two groups showed a difference in performance related to Time of assessment (pre- or post-training) on all the measures of cognitive functioning, while controlling for Age and Education.

Then, to better understand the specific effects of the training, we ran a series of repeated-measure 2 × 2 ANCOVAs on raw scores of each neuropsychological test, with Time of Assessment (2 levels: Pre-Training and Post-Training) as a within-subject variable, Group (2 levels: EG and CG) as the between-subject variable and Age and Education as covariates. In the case of a significant interaction, post-hoc analyses were performed (Duncan’s test for group comparison).

Finally, we analysed the effect of the training on the total number of neuropsychological tests whose performance was at ceiling (i.e. receiving an equivalent score of 4) for each participant of the two groups at both pre- and post-training assessment. To do so, a mixed ANOVA was performed on the sum of maximum equivalent scores, with Time of Assessment as within-subject and Group as between-subject factors.

## Results

### Pre-training

We did not find pre-existing differences between the two groups (EG and CG) before starting the training program, for Age (EG_mean score_ = 70.17, SD = 6.465, CG_mean score_ = 69.47, SD = 6.393; t_(81)_ = 0.474; *p* = 0.637), Education (EG_mean score_ = 11.87, SD = 3.758, CG_mean score_ = 10.68, SD = 3.857; t_(81)_ = 1.36; *p* = 0.171) and MoCA raw scores (EG_mean score_ = 25.77, SD = 2.144, CG_mean score_ = 25.21, SD = 2.29; t_(81)_ = 1.093; *p* = 0.278).

### Training effect

To investigate the overall effect of the training, a MANCOVA was performed on the raw scores of each test obtained at pre- and post-training sessions by the two groups, while controlling for the effects of Age and Education. Wilks’s lambda indicated the overall model was significant (F_(13,65)_ = 2.318, *p* = .013; η_p_^2^ = 0.317). Crucially, a significant three-way interaction between Tests, Time and Group (F_(13,1001)_ = 2.765, *p* = 0.001, η_p_^2^ = 0.035) revealed an overall impact of the training with the expected gain for the experimental group.

To further explore which of the cognitive domains were mainly influenced by the training program, 2 × 2 repeated-measure ANCOVAs were run for each neuropsychological test. First, the presence or absence of the interaction of interest (i.e. Time*Group) is reported. Then, the expected contribution of the covariates on the raw scores is also discussed (see Table 1 for a summary of the results).

**Table 1:** Summary of the significant results. Main effects, interactions and covariates resulted significant for each neuropsychological test

Test	Main and Interaction Effects			Covariates	
	Time	Group	Time * Group	Age	Education
MoCA	*p* < 0.01	n.s.	n.s.	*p* < 0.01	*p* < 0.01
Forward Digit Span	n.s.	n.s.	*p* = 0.051	*p* < 0.01	*p* < 0.01
Backward Digit Span	n.s.	n.s.	n.s.	*p* < 0.01	*p* < 0.01
Forward Corsi Span	n.s.	n.s.	n.s.	n.s.	n.s.
Backward Corsi Span	n.s.	n.s.	n.s.	*p* < 0.01	n.s.
Short Story Test	*p* < 0.01	*p* < 0.01	n.s.	*p* < 0.05	n.s.
Copy of ROCF	n.s.	n.s.	n.s.	*p* < 0.01	*p* < 0.01
Recall of ROCF	*p* < 0.01	n.s.	n.s.	*p* < 0.05	*p* < 0.05
Raven’s Matrices	n.s.	n.s.	*p* = 0.082	*p* < 0.01	*p* < 0.01
Semantic Verbal Fluency	n.s.	n.s.	*p* < 0.05	*p* < 0.01	*p* < 0.05
Phonological Verbal Fluency	n.s.	n.s.	n.s.	*p* < 0.01	*p* < 0.01
Stroop Test (time)	n.s.	n.s.	*p* < 0.01	*p* < 0.01	*p* < 0.05
Stroop Test (errors)	n.s.	n.s.	*p* < 0.05	n.s.	*p* < 0.05
TMT	*p* < 0.01	n.s.	n.s.	*p* < 0.01	*p* < 0.01

### Main and interaction effects

**Fig. 1:**
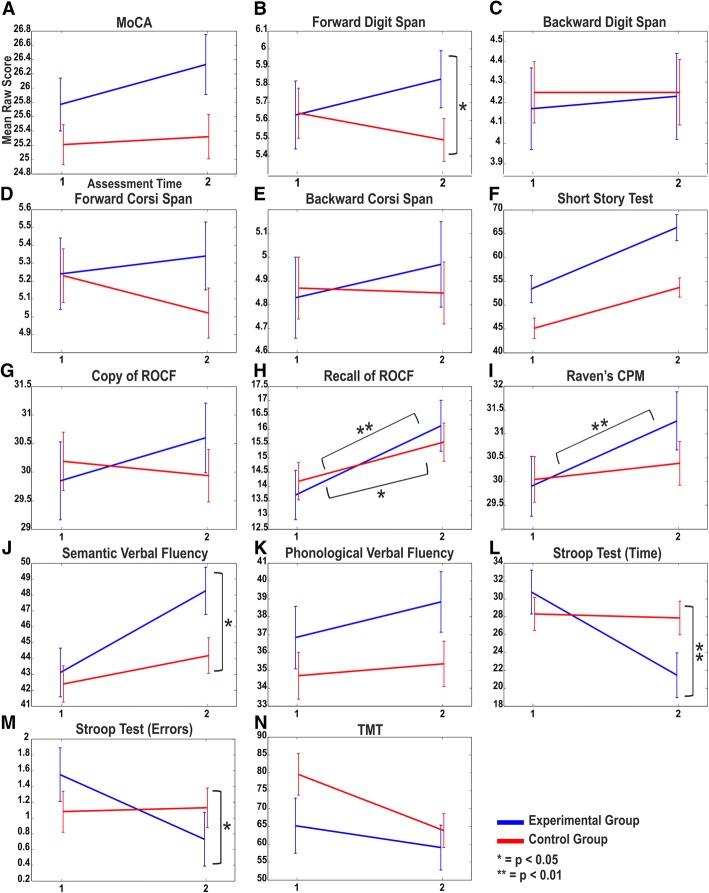
Neuropsychological assessment. Mean raw scores (and standard errors) at each neuropsychological test [**a** - **n**] for both Experimental (blue line) and Control group (red line) at pre- (1) and post-testing (2)

For the MoCA, Time of Assessment (F_(1,79)_ = 7.007, *p* < 0.01, η_p_^2^ = 0.081) was found to be significant, showing that the scores on the second assessment were higher than on the first assessment. Even though no significant interaction was found between Time and Group, a tendency of the EG to improve more than the CG over time can be observed (Fig. 1a).

For the Forward Digit Span, there was a nearly significant interaction between Time and Group (F_(1,79)_ = 3.914, *p* = 0.051, η_p_^2^ = 0.047). Post-hoc comparisons to investigate the interaction effect found no significant differences between conditions, nonetheless there is a trend level improvement of the EG, while the CG’s performance slightly worsened at the second assessment (Fig. 1b).

No significant main or interaction effects emerged in the Backward Digit Span (all ps > 0.05; Fig. 1c).

For the Forward Corsi Span, no main or interaction effect resulted to be significant (all ps > 0.05). Despite the absence of a significant interaction between Time and Group, from the graph it can be observed that at the second testing session while the EG slightly improved, the CG’s performance was reduced (Fig. 1d).

On the Backward Corsi Span, even though no main or interaction effect was observed (all ps > 0.05), a small improving trend was detected for the EG but not for the CG (Fig. 1e).

For the Short Story Test, there were significant effects for Group (F_(1,79)_ = 10.583, *p* < 0.01, η_p_^2^ = 0.104) and Time (F_(1,79)_ = 10.464, *p* < 0.01, η_p_^2^ = 0.130). The EG performed better than the CG in both assessments, and both groups significantly improved over time (Fig. 1f).

In the copy of ROCF, although no significant interaction between Time and Group was found, an improvement of the EG and a worsening of the CG can be qualitatively observed (Fig. 1g).

For the recall of the ROCF, Time of assessment (F_(1,79)_ = 10.492, *p* < 0.01, η_p_^2^ = 0.117) was significant. Both the EG and the CG recalled more elements at post-training testing, with the EG improving more than the CG as shown in the picture (Fig. 1h).

In Raven’s matrices, there was a trend level interaction effect between Time and Group (F_(1,78)_ = 3.106, *p* = 0.082, η_p_^2^ = 0.038). Exploratory post-hoc comparisons on the interaction effect revealed that the EG significantly improved after the training (*p* < 0.01), while no difference between the two assessments was found for the CG (Fig. 1i).

For the Semantic Verbal Fluency, the interaction between Time and Group (F_(1,79)_ = 5.454, *p* < 0.05, η_p_^2^ = 0.065) resulted to be significant. Post-hoc analyses to investigate the interaction effect showed that only the EG significantly (*p* < 0.01) improved over time (Fig. 1j).

In the Phonological Verbal Fluency, even if no cognitive training effect was found, the EG slightly improved over time, as compared to the CG (Fig. 1k).

In the time assessment of the Stroop Test, the ANCOVA identified a significant interaction between Time and Group (F_(1,79)_ = 7.666, *p* < 0.01, η_p_^2^ = 0.088). Post-hoc comparisons on the significant interaction indicated that only the EG resulted to be significantly (*p* < 0.01) faster at the second assessment, while no improvement was observed for the CG (Fig. 1l).

For the error assessment of the Stroop Test, the interaction between Time and Group (F_(1,79)_ = 4.232, *p* < 0.05, η_p_^2^ = 0.051) resulted significant. The post-hoc analyses to investigate the significant interaction showed that the EG, but not the CG, improved (*p* < 0.05) at the second assessment (Fig. 1m).

Finally, in the TMT, there was a significant effect for Time (F_(1,79)_ = 8.961, *p* < 0.01, η_p_^2^ = 0.102). Both groups improved over time (Fig. 1n).

### Covariate effects

Since we ran the ANCOVAS on the raw scores obtained by the participants, we expected significant effects of the covariates.

For the MoCA, the effects of Education (F_(1,79)_ = 7.652, *p* < 0.01, η_p_^2^ = 0.088), Age (F_(1,79)_ = 20.06, *p* < 0.01, η_p_^2^ = 0.203) and the interaction between Time and Age (F_(1,79)_ = 7.043, *p* < 0.01, η_p_^2^ = 0.082) were significant. More educated participants thus performed better than less educated, and, overall, younger participants obtained higher scores, especially to a greater extent in the post-training assessment.

For the Forward Digit Span, significant effects of Education (F_(1,79)_ = 10.47, *p* < 0.01, η_p_^2^ = 0.117) and Age (F_(1,79)_ = 5.425, *p* < 0.01, η_p_^2^ = 0.064) were observed. Younger and more educated subjects had a larger verbal span than older and less educated participants.

Education (F_(1,79)_ = 6.396, *p* < 0.01, η_p_^2^ = 0.075), Age (F_(1,79)_ = 7.353, *p* < 0.01, η_p_^2^ = 0.085) and the interaction between Time and Age (F_(1,79)_ = 4.35, *p* < 0.05, η_p_^2^ = 0.052) were significant for the Backward Digit Span. Overall, higher scores were related to higher education, while on the second assessment younger participants performed better than older subjects.

For the Backward Corsi Span the ANCOVA indicated a significant effect only of Age (F_(1,78)_ = 14.226, *p* < 0.01, η_p_^2^ = 0.154), with younger age predicting higher scores.

For the Short Story Test, there was a significant effect of Age (F_(1,79)_ = 6.432, *p* < 0.05, η_p_^2^ = 0.136) and the interaction between Time and Age (F_(1,79)_ = 8.003, *p* < 0.01, η_p_^2^ = 0.108). The interaction effect showed that, only during the second assessment, younger age was related to a greater number of recalled elements.

The ANCOVA on the copy of ROCF showed a significant effect of Education and Age (F_(1,79)_ = 8.097, *p* < 0.01, η_p_^2^ = 0.093 and F_(1,79)_ = 9.541, *p* < 0.01, η_p_^2^ = 0.108, respectively), revealing that younger and more educated subjects showed a better performance than older and less educated participants.

In the recall of ROCF, the effect of Education (F_(1,79)_ = 4.56, *p* < 0.05, η_p_^2^ = 0.055), Age (F_(1,79)_ = 6.659, *p* < 0.05, η_p_^2^ = 0.078) and the interaction between Time and Age (F_(1,79)_ = 7.453, *p* < 0.01, η_p_^2^ = 0.086) were significant. Overall, higher education was related to higher performance, while the interaction effect shows that younger participants recalled more elements than younger ones, but only during the second assessment.

For Raven’s matrices, there was a significant effect of Education (F_(1,78)_ = 14.423, *p* < 0.01, η_p_^2^ = 0.156) and Age (F_(1,78)_ = 19.101, *p* < 0.01, η_p_^2^ = 0.197), with, higher scores related to higher education and younger age.

For the Semantic Verbal Fluency test, both Education (F_(1,79)_ = 4.159, *p* < 0.05, η_p_^2^ = 0.050) and Age (F_(1,79)_ = 33.178, *p* < 0.01, η_p_^2^ = 0.296) were significant. Again, younger and more educated subjects performed better than older and less educated participants.

Also in the Phonological Verbal Fluency test there were significant effects for Education (F_(1,79)_ = 14.721, *p* < 0.01, η_p_^2^ = 0.157) and Age (F_(1,79)_ = 15.071, *p* < 0.01, η_p_^2^ = 0.160). Younger participants had higher scores, as higher education was related to a better performance.

In the time assessment of the Stroop Test, significant effects were identified for Education (F_(1,79)_ = 5.098, *p* < 0.05, η_p_^2^ = 0.061) and Age (F_(1,79)_ = 18.86, *p* < 0.01, η_p_^2^ = 0.193) Overall, younger and more educated participants were faster than older and less educated subjects.

For the error assessment of the Stroop test, only Education (F_(1,79)_ = 4.346, *p* < 0.05, η_p_^2^ = 0.052) was significant. More educated participants made fewer errors than less educated ones.

Finally, for the TMT, the effect of Education (F_(1,79)_ = 7.439, *p* < 0.01, η_p_^2^ = 0.086), Age (F_(1,79)_ = 20.725, *p* < 0.01, η_p_^2^ = 0.208) and the interaction between Time and Age (F_(1,79)_ = 13.708, *p* < 0.01, η_p_^2^ = 0.148) were found to be significant. Overall, more educated participants performed better than less educated ones, and younger subjects were faster but only during the second assessment.

### Sum of maximum equivalent scores

**Fig. 2:**
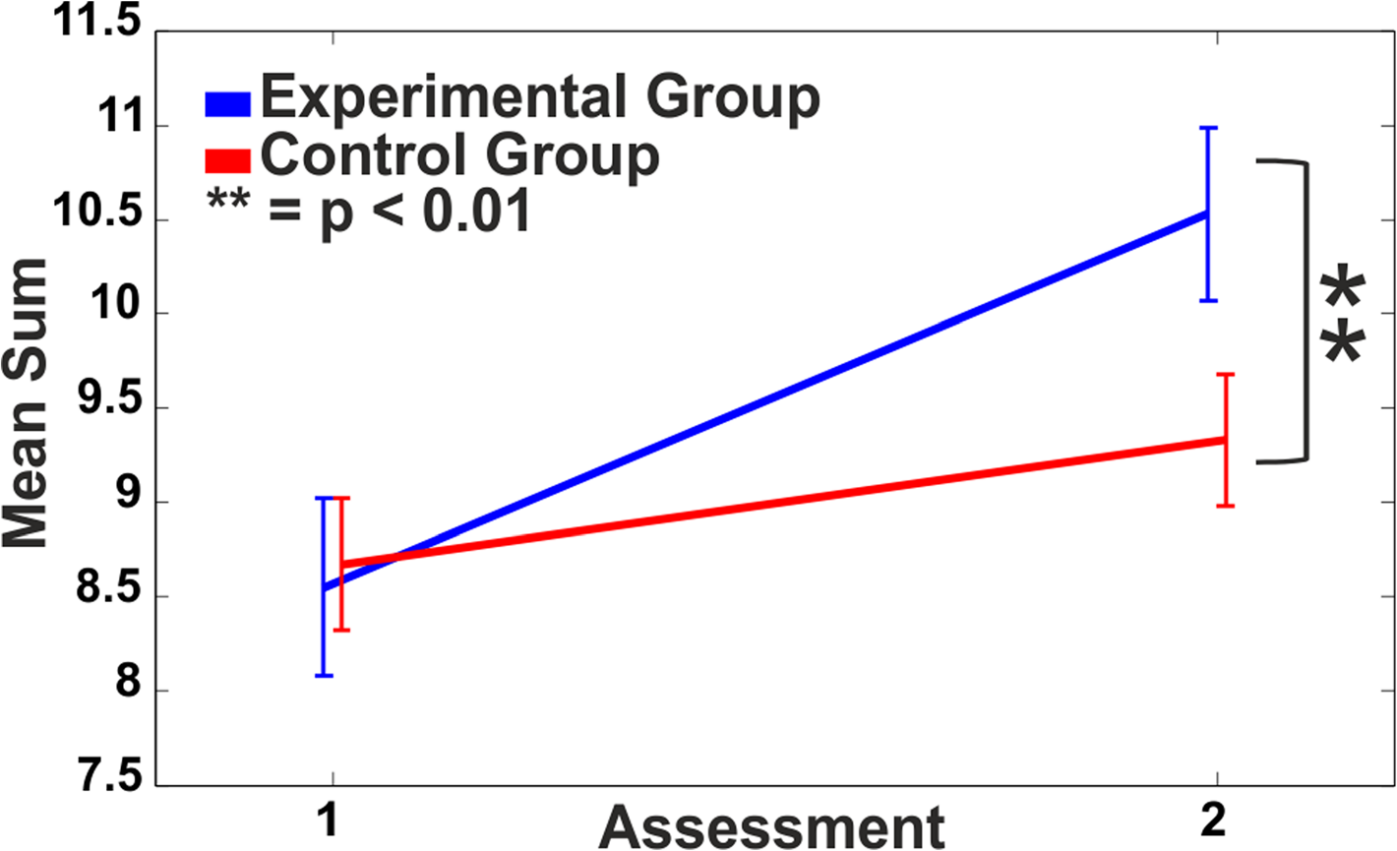
Numerosity of maximum equivalent scores. Mean sum of neuropsychological tests receiving an equivalent score of 4 for both Experimental (blue line) and Control Group (red line) at pre- (1) and post-training (2)

The mixed ANOVA on the sum of maximum equivalent scores revealed a significant interaction between Time and Group (F_(1,81)_ = 7.19, *p* < 0.01, η_p_^2^ = 0.08). Post-hoc analyses showed that there was a significant increase (*p* < 0.001) in the number of tests reaching ceiling level only for the EG, as compared to the CG (Fig. 2).

### Post-training questionnaire

**Fig. 3:**
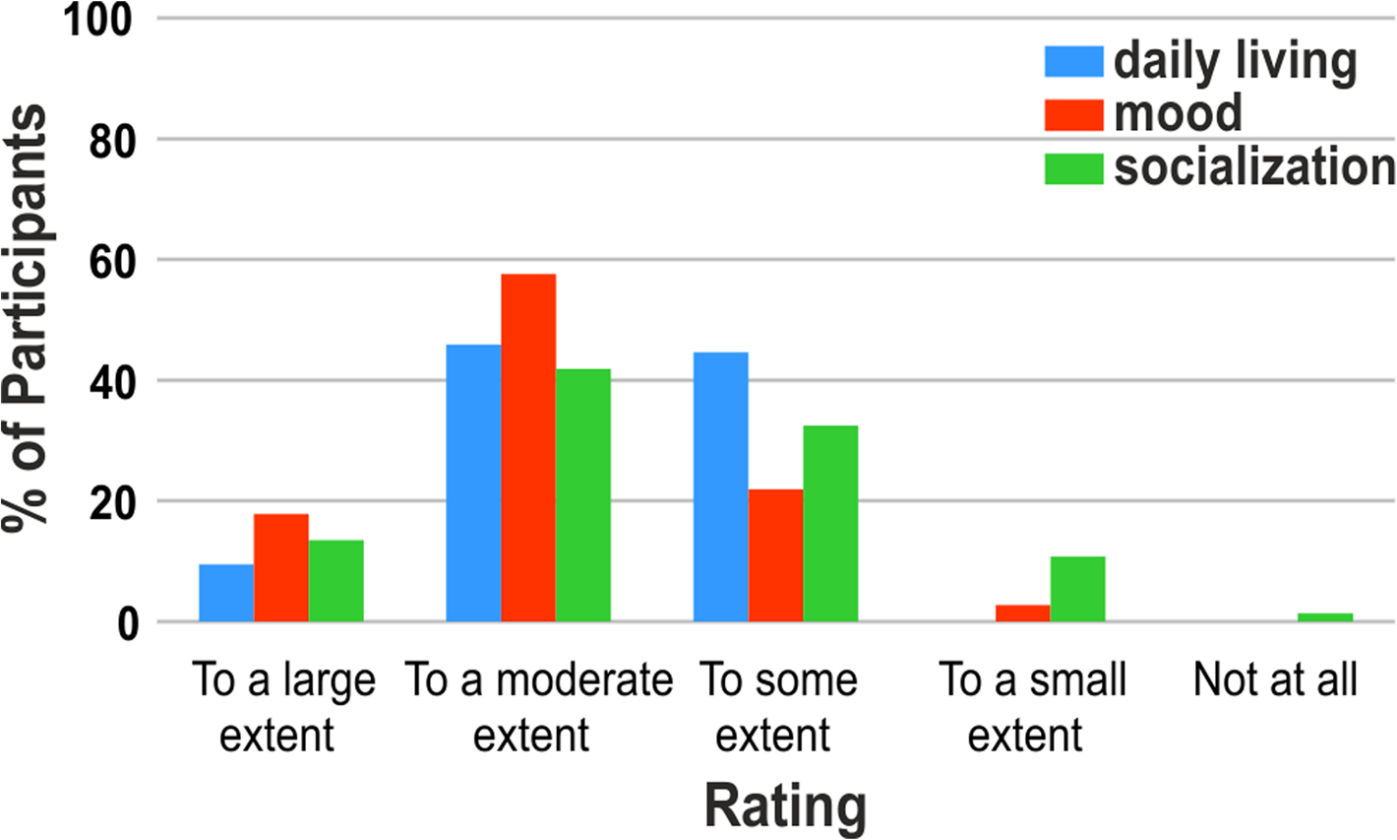
Training effects (Questionnaire). Impact of the training on daily living, mood, and socialization as assessed by a questionnaire after the training

To investigate the effect of the training on everyday life aspects a questionnaire was administered to the participants at the end of the program. As it can be observed from the graph (Fig. 3), the majority of the participants subjectively perceived a positive influence of the training on their daily living functioning, on their mood, and also on their socialization (rating ‘To a large extent’: 9.46% for daily living, 17.81% for mood and 13.51% for socialization. Rating ‘To a moderate extent’: 45.95% for daily living, 57.53% for mood and 41.89% for socialization. Rating ‘To some extent’: 44.59% for daily living, 21.92% for mood and 32.43% for socialization).

## Discussion

The study, at least to our knowledge, is one of the first multi-domain interventions were individual cognitive differences are taken into account in order to provide the older participants with an optimal amount of mental stimulation. It indeed confirms the effectiveness of a short multi-domain cognitive training [26, 31] in a group of healthy elderly aged over 60 years, highlighting how promising results can be achieved also with a low-intensity commitment of one session per week.

Specifically, the analyses suggested that the effect of the training program was not comparable across all cognitive domains. Greater improvements could be observed on executive functions (as measured by Stroop and Semantic Verbal Fluency tests) and short term memory (as measured by Forward Digit Span test). Even general cognitive functioning, as assessed by the MoCA, and non-verbal reasoning, as assessed by the Raven’s test, showed a tendency to an improvement only in the experimental group. Finally, the experimental group also qualitatively exhibited a slight enhancement in the other cognitive domains post-training, except for visuo-spatial and long-term memory tests that seemed to be unaffected by the engagement in a training program. Thus, it seems like “frontal lobe” functioning mainly benefited from this cognitive training program, aligning with the outcomes of previous studies employing higher-intensity multi-domain interventions (e.g. [26, 31]). No major improvement in attention was instead evident, as compared to the training intervention of Binder and colleagues [27]. Such a result might be due to the intrinsic nature of the ecological exercises proposed, since they were designed with the aim to stimulate higher cognitive processes (e.g. planning, reasoning) or complex interactions among different skills (e.g. memory, visuo-spatial abilities). This result appears to be a promising outcome for healthy older adults as executive functioning is reported to decrease with age (see [45] for a review), and because of its relevance in everyday life activities [46].

As previously demonstrated, the covariates, age and years of education, had a significant effect on many cognitive domains. In general, we observed that younger and more educated individuals performed better than older and less educated subjects, as expected.

The fact that we did not find large and generalised effects of the training on all the cognitive domains investigated could be due to different reasons. First, the low intensity of the training, both in terms of sessions per week (one) and total length of the program (~ 3 months) may have been insufficient to generate significant impact across all cognitive domains. The comparison with a more intense training dosage or with a longer training could verify its impact in future studies. Another potential explanation for our findings relates to the level of improvement attainable by a healthy elderly population. It is possible that in one or more cognitive domains each participant was already functioning at ceiling, thus leaving little room for improvement. In order to verify this hypothesis, we analysed the effect of the training by computing the total number of neuropsychological tests whose performance was at ceiling for each participant of the two groups. As hypothesized, a significant increase in the number of tests reaching ceiling level was evident only for the experimental group, as compared to the control group. Thus, this result seems to justify the reduced training effects observed, but at the same time highlights the possibility that the cognitive performance can be increased in the healthy population.

The effect of the training was not only measured through objective performance on cognitive tasks, but also assessed by means of a questionnaire where participants were required to rate the subjectively perceived influence of the cognitive stimulation on everyday life aspects. Even if subjective in its nature, relevant information could still be collected through this questionnaire, given also that many trials do not provide any kind of functional outcome measure (either subjective or objective; see [13] for a review). Having an impact on daily living functioning is indeed the ultimate goal that every training intervention should achieve in order to be really effective. However, measuring everyday functioning is made difficult by the absence of appropriate protocols that either are too simple to assess healthy subjects (e.g. IADL: Instrumental Activities of Daily Living; [47]) or cannot be fully administered to populations living in different countries (e.g. EPT: Everyday Problems Test; [48]). The choice of a questionnaire was thus driven by the lack of a testing protocol specific to the Italian population. Following training, the majority of the participants reported to have subjectively experienced an improvement in their everyday life and a positive influence on both mood and socialization. While the effect on daily living might be due to the cognitive stimulation, the social nature of the program, with sessions performed in small groups, might have had a greater influence on the more psychological dimensions of mood and socialization. These subjective reports are in line with findings of increased self-efficacy [49] that in turns improves the effects of cognitive interventions [50] experienced in a group setting. Being socially involved might act as a powerful motivational boost that encourages the elderly to fully commit to the training.

Some limitations of the present study must be taken into account. First of all, we are not able to dissociate the effect of the type of training from the effect of being in a group for a three-month period; moreover, no actual follow-up was performed after the end of the training in order to test whether the observed improvements were maintained over time. Second, the allocation to the experimental or control group was not random, as participants were assigned to each group based on their time of application to the program. Third, as not all the participants reached the 85% attendance, we cannot fully exclude that those not attending all the minimum required sessions were those who did not show training-related gains, thus somehow selecting themselves out (note however that none of them dropped out of the program). On the same line, despite the low intensity of the program, these participants raise the issue of feasibility of constantly attending the training. Finally, the post-training questionnaire is a subjective measure of training transfer to everyday living, and not an objective measure of functional outcome. However, taken together, our results show that even a low-intensity training program can promote some of those protective factors (engagement in demanding intellectual activities, social involvement, maintenance of an appropriate mood level) that are reported [9, 10] to support successful cognitive ageing. Moreover, a multi-domain approach confirms to be an excellent training method (see [27, 28]) to transfer gains not only to other cognitive domains (as assessed by neuropsychological tests), but also to everyday living (as subjectively reported by participants). Although larger improvements might be achieved by increasing the number of sessions, this training intensity appears to be affordable both in terms of costs and resources (neuropsychologists) employed and participants’ commitment and compliance. Notably, commitment and compliance can be obtained if individual differences are taken into account, in order to have appropriately demanding requests and avoid frustration.

## Conclusion

Our data support the idea that cognitive functioning in healthy older adults can benefit from cognitive training interventions [13, 14]. We showed that, despite the low intensity of the training and the high performance of healthy older individuals, the training proposed was effective in improving the subjective perception of everyday compliance and the objective performance at cognitive tests. In a world where any significant cure for neurodegenerative diseases is yet to be determined, engaging in stimulating cognitive activities might promote positive brain plasticity, in order not only to contrast physiological ageing, but also eventually and hopefully delay the clinical manifestation of dementia.

## Additional file

Additional file 1: Post-training questionnaire. Original questionnaire (in Italian) and English translation. (DOCX 20 kb)

## Abbreviations

CG: Control Group
CPM: Coloured Progressive Matrices
EG: Experimental Group
MoCA: Montreal Cognitive Assessment
ROCF: Rey-Osterrieth Complex Figure
TMT: Trail Making Test
