# Supplementary material for: A group study on the effects of a short multi-domain cognitive training in healthy elderly Italian people
Source: BMC Geriatr. 2018 Dec 27;18:321. doi: 10.1186/s12877-018-1014-x (PMC6307149; doi:10.1186/s12877-018-1014-x)
Supplement: Supplementary file 1 — Post-training questionnaire. Original questionnaire (in Italian) and English translation. (DOCX 20 kb) [file 12877_2018_1014_MOESM1_ESM.docx]

**QUESTIONARIO DI VALUTAZIONE DEL CORSO DI POTENZIAMENTO COGNITIVO [ITALIAN]**

**1) Le sono piaciuti gli esercizi proposti durante il corso?** moltissimo  molto  sufficientemente  poco  per niente

**2) Ha trovato interessanti gli interventi informativi (su tematiche quali memoria, attenzione, riserva cognitiva, alimentazione etc…) condotti durante il corso?** moltissimo  molto  sufficientemente  poco  per niente

**3) Ha trovato adeguati gli strumenti e i materiali impiegati (proiettore, schede di risposta…)?** moltissimo  molto  sufficientemente  poco  per niente

**4) Ha trovato adeguata la modalità di conduzione del corso da parte della psicologa?** moltissimo  molto  sufficientemente  poco  per niente

**5) Sente che gli interventi informativi abbiamo migliorato le sue conoscenze in merito alle sue capacità cognitive e alle altre tematiche trattate (quali, ad esempio, la riserva cognitiva, l’alimentazione etc..)?**

 moltissimo  molto  sufficientemente  poco  per niente

**6) Sente che la frequentazione del corso abbia avuto delle ripercussioni positive nella vita quotidiana?**

 moltissimo  molto  sufficientemente  poco  per niente

**7) Sente che la frequentazione del corso abbia avuto un impatto positivo sul suo umore?** moltissimo  molto  sufficientemente  poco  per niente

**8) Sente che la frequentazione del corso le abbia dato la possibilità di conoscere persone nuove, coltivare nuove relazioni e di conseguenza abbia avuto un impatto positivo sugli aspetti di socializzazione?**

 moltissimo  molto  sufficientemente  poco  per niente

**9) Pensa che anche in futuro potrebbe essere interessato/a a frequentare un corso di questo tipo?**

 moltissimo  molto  sufficientemente  poco  per niente

**10) Consiglierebbe alle altre persone la frequentazione di un corso di questo tipo?** moltissimo  molto  sufficientemente  poco  per niente

**11) Pensa che durante gli incontri individuali di valutazione NON siano stati indagati alcuni aspetti relativamente ai quali ha riscontrato un miglioramento grazie alla frequentazione del corso?**

 Sì  No

**Se sì, quali?** ………………………………………………………………………………………………………………………………………………………………………………………………………………………………………………………………………………………………………………………………………………………………………………………………………………………………………………………………………………

**12) Indichi quali sono stati i punti di forza del corso:**

………………………………………………………………………………………………………………………………………………………………………………………………………………………………………………………………………………………………………………………………………………………………………………………………………………………………………………………………………………

**13) Indichi quali sono stati i punti di debolezza del corso:**

………………………………………………………………………………………………………………………………………………………………………………………………………………………………………………………………………………………………………………………………………………………………………………………………………………………………………………………………………………

**14) Spazio dedicato a consigli/suggerimenti/altre considerazioni:**

………………………………………………………………………………………………………………………………………………………………………………………………………………………………………………………………………………………………………………………………………………………………………………………………………………………………………………………………………………

**EVALUATION QUESTIONNAIRE OF THE COGNITIVE TRAINING PROGRAM [ENGLISH]**

**1) Did you like the exercises proposed during the training?**

 a lot  very much  enough  little  not at all

**2) Have you found the informative interventions (on memory, attention, cognitive reserve, diet, etc…) proposed during the training interesting?**

 a lot  very much  enough  little  not at all

**3) Do you think that the tools and materials used were appropriate (projector, answer sheets…)?**

 a lot  very much  enough  little  not at all

**4) Do you think that the psychologist conducted the training appropriately?**

** a lot  very much  enough  little  not at all**

**5) Do you think that the informative interventions improved your knowledge about your cognitive abilities and all the other topics discussed (e.g., cognitive reserve, diet, etc…)?**

 a lot  very much  enough  little  not at all

**6) Do you think that attending the training had positive consequences in your daily life?**

 a lot  very much  enough  little  not at all

**7) Do you think that attending the training had a positive impact on your mood?**

 a lot  very much  enough  little  not at all

**8) Do you think that attending the training allowed you to meet new people, grow new relationships and, as a consequence, had a positive impact on your socialization?**

 a lot  very much  enough  little  not at all

**9) Do you think that in the future you will be interested in attending another similar training?**

 a lot  very much  enough  little  not at all

**10) Would suggest other people attend a similar training?**

 a lot  very much  enough  little  not at all

**11) Do you think that during the individual assessments some aspects where you perceive an improvement thanks to the training were not investigated?**

 Yes  No

**If yes, which ones?**

………………………………………………………………………………………………………………………………………………………………………………………………………………………………………………………………………………………………………………………………………………………………………………………………………………………………………………………………………………

**12) Write down the strengths of the training:**

………………………………………………………………………………………………………………………………………………………………………………………………………………………………………………………………………………………………………………………………………………………………………………………………………………………………………………………………………………

**13) Write down the weaknesses of the training:**

………………………………………………………………………………………………………………………………………………………………………………………………………………………………………………………………………………………………………………………………………………………………………………………………………………………………………………………………………………

**14) Other advice/suggestions/comments:**

………………………………………………………………………………………………………………………………………………………………………………………………………………………………………………………………………………………………………………………………………………………………………………………………………………………………………………………………………………
